# Rarely Observed Histopathological Subtypes of Endometrial Malignant Tumors in a Tertiary Care Center: A Case Series

**DOI:** 10.7759/cureus.70282

**Published:** 2024-09-26

**Authors:** Trisha Sankaran, Sandhya Sundaram, Divya Dhanabal, Arumugam Vasugi, Pavithra V, Lawrence D'Cruze

**Affiliations:** 1 Pathology, Sri Ramachandra Institute of Higher Education and Research, Chennai, IND; 2 Pathology, Sri Ramachandra Medical College and Research Institute, Chennai, IND

**Keywords:** carcinosarcoma of the uterus, endometrial biopsy, endometrial carcinoma, endometrial stromal cell sarcoma, mesonephric like adenocarcinoma, mmr deficient, postmenopausal bleed

## Abstract

Introduction

Endometrial cancer, one of the most prevalent gynecological malignancies, represents a significant contributor to global mortality and morbidity. The histological subtype of this cancer is pivotal in treatment planning and patient risk stratification. This case series, comprising seven cases, underscores the significance of rare endometrial cancer variants and the importance of ancillary studies, specifically immunohistochemistry, in comprehending and diagnosing the condition.

Materials and methods

The data of all the rare histological subtypes of malignant endometrial tumors encountered from April 2021 to April 2024 were collected. The data collected included age; risk factor; histological type; tumor, node, metastasis (TNM) stage; the International Federation of Gynecology and Obstetrics (FIGO) grade; P53; and mismatch repair (MMR) status. The histopathology specimens were reviewed using a Labomed microscope (LX500) manufactured in India. Images were taken and added wherever necessary, and the results were studied, analyzed, clinically correlated, and are shown in tables.

Results

Seven rare histopathological subtypes of malignant endometrial tumors were encountered in this period in this tertiary care center. This included mesonephric like adenocarcinoma, high-grade carcinosarcoma, carcinosarcoma with the serous and chondrosarcomatous components, clear cell carcinoma of endometrium, endometrial stromal sarcoma with smooth muscle differentiation, endometrial stromal sarcoma, and undifferentiated sarcoma with focal myxoid degeneration.

Conclusion

Diagnosis of uncommon endometrial carcinomas proves to be challenging due to their various histological subtypes, most often with overlapping features. The cornerstone of effective care lies in collaboration with clinicians, radiologists, and pathologists to identify these lesions as early as possible using a multidisciplinary approach for optimal patient outcome management.

## Introduction

Endometrial cancer is the most prevalent gynecological malignancy and is a significant cause of mortality and morbidity worldwide [1]. Due to its clinical relevance and to risk stratify the patients, the histological subtype plays a pivotal role in treatment planning. This case series of seven cases emphasizes the rare variants and the importance of ancillary studies, such as immunohistochemistry (IHC), in understanding and making a diagnosis. Broadly, we can divide endometrial carcinomas into two main histological types: endometrioid endometrial carcinomas (EEC) and non-endometrioid endometrial carcinomas (NEEC). EEC is the most commonly seen and comprises 75%-80% of the tumors having a favorable prognosis. NEEC includes endometrial serous carcinoma, clear cell carcinoma, carcinosarcoma, squamous cell carcinoma, dedifferentiated carcinoma, undifferentiated carcinoma, mesonephric-like adenocarcinoma, and mixed tumor subtypes. These constitute the remaining 20%-25% of the tumors and are known to have a poor prognosis due to their aggressive behavior [2].

## Materials and methods

This case series consists of seven cases of rare histological subtypes of endometrial carcinomas diagnosed by the Department of Pathology, Sri Ramachandra Institute of Higher Education and Research, which is a tertiary care hospital based in Southern India. 

Endometrial biopsies and hysterectomy specimens of patients who presented with chief complaints of post menopausal bleeding and thickened endometrium, sent by the Department of Obstetrics and Gynecology of Sri Ramachandra Institute of Higher Education and Research, were considered for the case series. Once the rare subtypes were identified, the data of these cases were collected after getting informed consent from the respective patients.

The data collected included age; risk factor; histological type; tumor, node, metastasis (TNM) stage; the International Federation of Gynecology and Obstetrics (FIGO) grade; P53; and mismatch repair (MMR) status. The findings of the histopathology specimens were studied and documented using a Labomed microscope (LX500) manufactured in India. Ancillary testing done for the cases were also documented. Images of the tumor morphology and the IHC studies done were taken and added wherever necessary. The results were studied, analyzed, clinically correlated, and are shown in tables.

Inclusion criteria

The study included all rare histological subtypes of endometrial carcinoma diagnosed from hysterectomy specimens in 2021-2024.

Exclusion criteria

This case series excluded cases with only endometrial biopsies, a lack of suitable tissue blocks, insufficient or incomplete medical records, and patients lost to follow-up. Benign conditions diagnosed were also excluded from the data collection.

## Results

Case 1

A 60-year-old postmenopausal woman presented with complaints of bleeding per vaginum and fatigue for the past two weeks. She is also a known case of type 2 diabetes mellitus and systemic hypertension for the past seven years, on regular oral medications (Table 1). She has two children, and her past surgical history revealed a tubectomy. On evaluation, ultrasonography revealed a uterus measuring 8 x 3 x 4.3 cm, having an endometrial thickness of 6.1 mm and hyperechogenic. Given thickened endometrium, she underwent a hysteroscopic endometrial biopsy. 

**Table 1:** Summary of the rare histological subtypes of endometrial carcinoma ER: Estrogen receptor; PR: progestrone receptor; CEA: carcinoma embryonic antigen; SMA: smooth muscle actin; MMR: mismatch repair

S. no	Case summary	Microscopy	Ancillary testing
1.	A 60-year-old multiparous woman presents with vaginal bleeding and has a medical history of type 2 diabetes mellitus and systemic hypertension	Infiltrating tumor arranged in small tubules, nests, and sheets. Tubules with intraluminal secretions. Cells of moderate nuclear pleomorphism and vesicular chromatin	GATA3 and PAX8-positive. P53-wild type, Cyclin D1-negative, P16-non-contributory, MMR-deficient
2.	A 60-year-old nulliparous, retrovirus positive presenting with fever and abdominal pain and has a medical history of systemic hypertension, type 2 diabetes mellitus, and hypothyroidism	Biphasic morphology, tumor cells in vague glandular pattern and intervening stroma of pleomorphic spindled cells. Giant tumor cells present as well as focal cartilaginous differentiation	-
3.	A 59-year-old woman multiparous woman with a medical history of systemic hypertension and hypothyroidism. She is currently experiencing fever and abdominal pain, as well as post-menopausal bleeding	Biphasic morphology Carcinomatous elements (serous carcinoma) admixed with sarcomatous elements (chondrosarcoma)	P16-strong diffuse positivity in the serous component
4.	A 66-year-old multiparous woman presented with postmenopausal bleeding. Also has systemic hypertension, dyslipidemia, and type 2 diabetes mellitus	Round to polygonal cells with abundant clear cytoplasm. Focal areas of cells with eosinophilic cytoplasm and hobnailing	Napsin A, Vimentin, and CK7-positive. ER, PR, CEA were negative in the tumor cells. P53-mutant type, MMR-proficient
5.	A 52-year-old multiparous postmenopausal woman presented with foul smelling discharge	Nests of tumor cells, spindled morphology, coarse chromatin, scattered mitotic figures	Cyclin D1, SMA, and Desmin-positive; Ki67-60%
6.	A 47-year-old multiparous woman presented with abnormal uterine bleeding	Sheets of tumor cells. spindling of cells, indistinct cell borders, vesicular nuclei	CD10-positive, Cyclin D1-negative, Ki67-50%
7.	A 67-year-old multiparous woman presented with postmenopausal bleeding	Sheets of tumor cells, epitheloid morphology, focal myxoid degeneration	TLE, CD10, and Vimentin-positive; Ki67-90%

**Figure 1:**
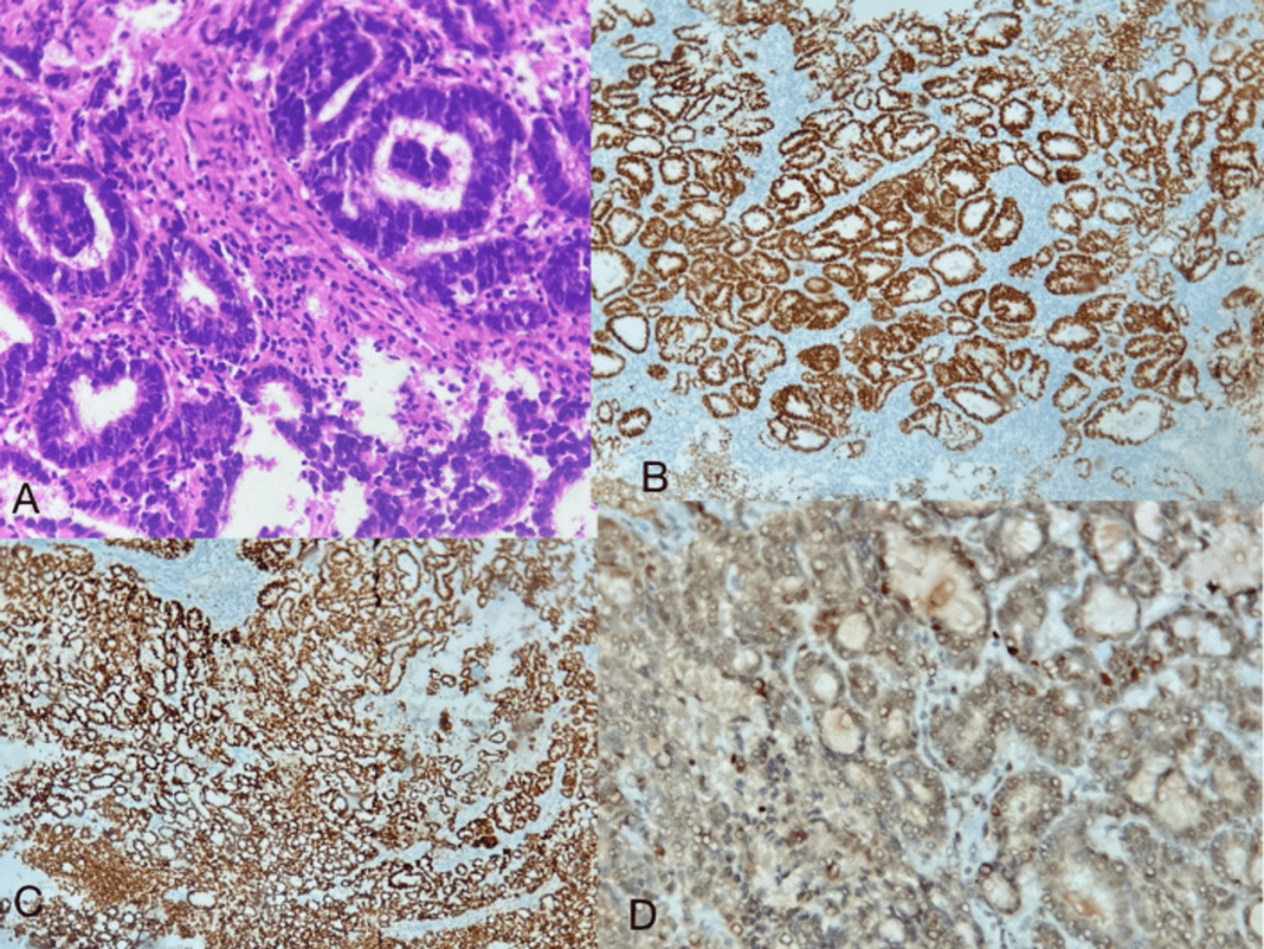
Mesonephric-like adenocarcinoma H&E: Hematoxylin and eosin; IHC: immunohistochemistry (A) Tumor cells arranged in tubules (20x magnification) (H&E). (B) PAX8 shows diffuse positivity in tumor cells (IHC). (C) GATA3 showing diffuse positivity in the tumor cells (IHC). (D) P16 staining was non-contributory (IHC)

Endometrial biopsy showed endometrial tissue with an infiltrating neoplasm composed of tumor cells arranged in tubules, sheets, and nests. Many of the tubules showed intraluminal secretions. The individual tumor cells are round to oval shaped with increased nuclear-cytoplasmic ratio, moderate nuclear pleomorphism, vesicular nuclear chromatin, and moderate to scant eosinophilic cytoplasm. The diagnosis of endometrial carcinoma was confirmed. Due to its distinct morphology, the mesonephric-like adenocarcinoma subtype was suspected. Consequently, immunohistochemical analysis was conducted to substantiate the diagnosis. By IHC, the tumor cells were diffusely positive for GATA3 and PAX8. P53 was of the wild type. Tumor cells were negative for Cyclin D1, and P16 was non-contributory (Figure 1). Based on histology and IHC findings, a diagnosis of mesonephric-like adenocarcinoma of the endometrium was given.

**Figure 2:**
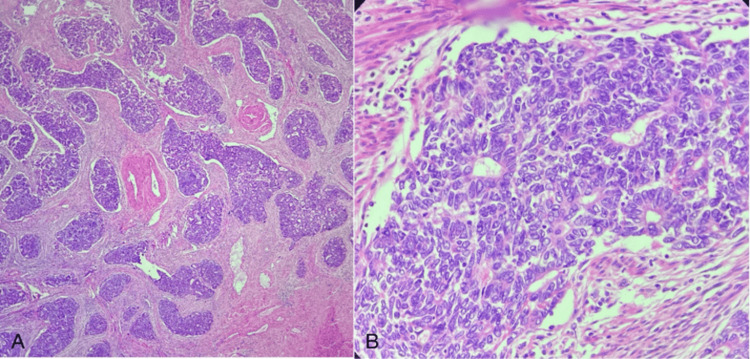
Mesonephric-like adenocarcinoma (resection specimen) H&E: Hematoxylin and eosin (A) Resection specimen showing an infiltrating tumor arranged in sheets and tubules (10x magnification) (H&E). (B) Individual tumor cells show an increased N:C ratio, having vesicular chromatin (40x magnification) (H&E)

Following this, the patient underwent a staging laparotomy. On gross examination, a grey-white friable tumor was noted in the uterus. The tumor was seen invading the myometrium to about 90% of the total myometrial thickness. On microscopic examination, the tumor morphology was consistent with the biopsy findings, and it was seen 90% infiltrating into the myometrium (Figure 2). Cervical stroma infiltration was not seen. Bilateral fallopian tubes and ovaries showed no abnormalities. Forty-four lymph nodes were examined, and they were free of tumor. Peritoneal wash sent for cytological examination showed only reactive mesothelial cells, and no malignant cells were seen. With these findings and correlating with the biopsy reports, a final diagnosis of endometrial mesonephric-like adenocarcinoma, high grade-pT1b pN0 (according to the American Joint Committee on Cancer (AJCC) 8th edition) was assigned, and a FIGO grade IIC was given due to this aggressive histological subtype. Immunohistochemical analysis for mismatch repair proteins was done, which showed that she was MMR-deficient and that methylation of the MLH1 promoter could be done to further evaluate germline/somatic mutations.

Case 2

A 60-year-old nulliparous postmenopausal woman came with complaints of fever of more than 100°F and a dull aching pain. She was diagnosed with systemic hypertension, hypothyroidism, and type 2 diabetes mellitus 10 years ago and is on regular oral medication for the same (Table 1). She is also retrovirus positive and has been on antiretroviral therapy for the past 22 years (tenofovir, lamivudine, and dolutegravir (TLD) regimen). Ultrasound showed a lesion arising from the body of the uterus measuring 4 cm in dimension, following which she underwent a hysteroscopic endometrial biopsy, which revealed carcinosarcoma of the endometrium. Staging laparotomy was done.

**Figure 3:**
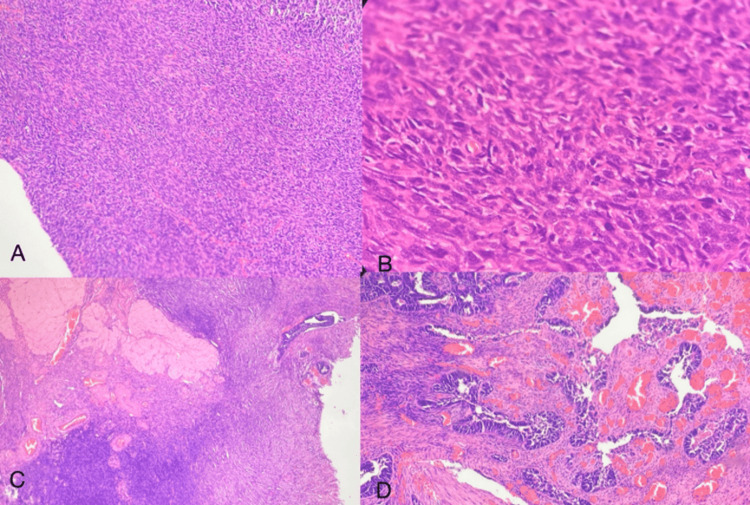
High-grade carcinosarcoma H&E: Hematoxylin and eosin (A) Diffusely infiltrating malignant neoplasm arranged in sheets (4x magnification) (H&E). (B) The Individual cells have a spindled morphology with moderate nuclear pleomorphism (20x magnification) (H&E). (C and D) Ovarian parenchyma showing involvement by the tumor (H&E)

Grossly, a grey-white friable lesion was noted arising from the body of the uterus and extending into the lower uterine segment with 80% myometrial invasion. A high-grade biphasic malignant neoplasm was noted on microscopic examination, composed of tumor cells arranged in sheets and a focal glandular pattern. The individual cells showed moderate nuclear pleomorphism, irregular nuclear membrane, vesicular nuclear chromatin with prominent nucleoli, and scant eosinophilic cytoplasm. The intervening stroma was cellular and was composed of pleomorphic spindle-shaped cells (Figure 3). Atypical mitotic figures, numerous giant tumor cells, and focal areas of cartilaginous differentiation were noted. The right ovary and right pelvic node showed tumor infiltration. The left ovary, bilateral fallopian tubes, omentum, and left pelvic node showed no evidence of tumor deposits. The peritoneal wash sent for cytology was negative for malignant cells. With all the above findings, a final diagnosis of high-grade carcinosarcoma, pT3b (parametrial involvement) pN1a (according to AJCC 8th edition), and FIGO stage IIIC1i (micrometastasis to pelvic node) was given. 

Case 3

A 59-year-old female presented with complaints of fever, vague pain in the abdomen, and on and off postmenopausal bleeding for the last six months (Table 1). She has an obstetric history of three children, and the last child was born 30 years ago, during which sterilization was also done. She is on regular oral medications for systemic hypertension and hyperthyroidism. She underwent a coronary artery bypass graft procedure for coronary artery disease a few years back. All baseline investigations were done, and she was found to be anemic, having a hemoglobin of 10.2g/dL. All other parameters were under normal limits. MRI showed increased endometrial thickness, suspicious of malignancy, for which staging laparotomy with radical hysterectomy and bilateral salpingo-oophorectomy along with omentectomy and pelvic node debulking surgery was done. 

**Figure 4:**
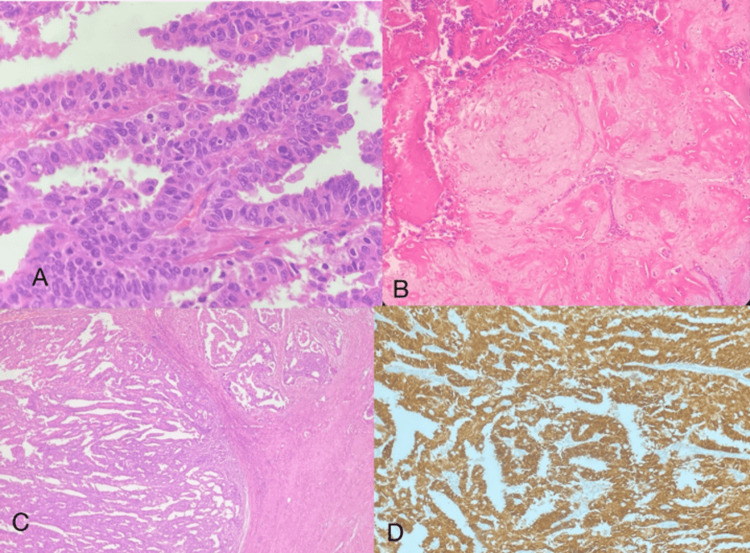
Carcinosarcoma H&E: Hematoxylin and eosin; IHC: immunohistochemistry (A-C) Malignant biphasic cell proliferation of carcinomatous element admixed with sarcomatous element (chondrosarcoma in B) (H&E). (D) P16 showed strong nuclear and cytoplasmic positivity in the carcinomatous area, confirming the serous component (IHC)

A grey-white lesion was seen in the endometrium with a myometrial invasion of less than 50% on gross examination of the resected specimen. Bilateral fallopian tubes and ovaries appeared unremarkable. On microscopic examination, sections from the endometrial lesion showed a biphasic malignant infiltrating neoplasm composed of tumor cells arranged in a papillary pattern with individual cells showing scant eosinophilic cytoplasm, nuclear hyperchromasia, prominent nucleoli, and frequent mitotic figures with atypical mitosis. This was representative of the carcinomatous elements (serous carcinoma). Adjacent areas also showed abundant cartilaginous matrix with few chondrocytes and myxoid degeneration. This confirmed the sarcomatous elements (chondrosarcoma). The lower uterine segment was involved along with the cervical stroma. Nine out of 35 lymph nodes sampled were showing tumor deposits. Bilateral fallopian tubes, ovaries, and the omentum showed no evidence of tumor. Due to the biphasic morphology, a diagnosis of carcinosarcoma was made. IHC for p16 was also done to confirm the serous component (Figure 4). A total of 90% of the tumor cells showed strong nuclear and cytoplasmic positivity in the carcinomatous element, proving the serous carcinoma. According to AJCC 8th edition, a stage of pT2pN1a was given along with FIGO stage IIIC1.

Case 4

A 66-year-old female presented with complaints of postmenopausal bleeding. She attained menopause 17 years ago (Table 1). Obstetric history revealed that she had three children, with the last childbirth at 40 years of age, and a sterilization procedure was done during the same time. She has been on regular oral medications and insulin injections for systemic hypertension, dyslipidemia, and type 2 diabetes mellitus for the last 10 years. Transvaginal ultrasound showed a thickened endometrium measuring 13.7 mm along with a bulky uterus. She was posted for staging laparotomy with radical hysterectomy and bilateral salpingo-oophorectomy procedure in view of a high suspicion of malignancy. 

**Figure 5:**
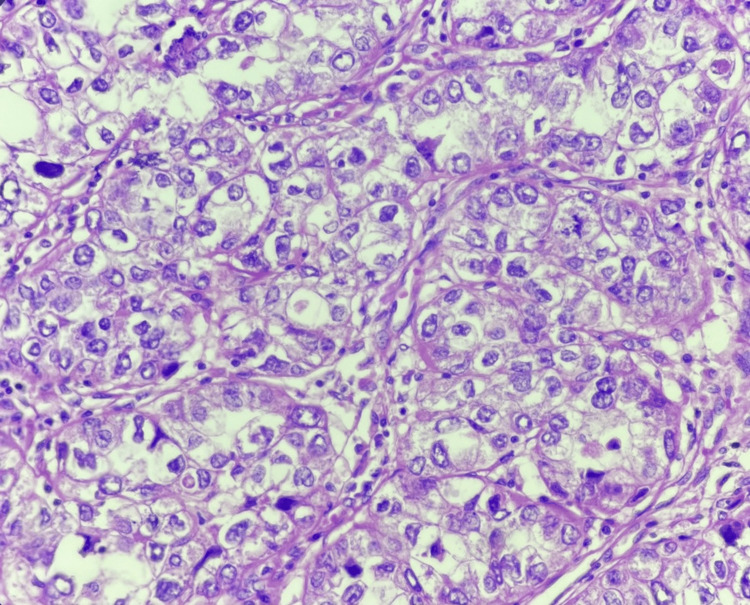
Clear cell carcinoma H&E: Hematoxylin and eosin Round to polygonal cells with mild nuclear pleomorphism and having abundant clear cytoplasm (40x magnification) (H&E)

On gross examination, a grey-white papillary lesion was noted at the uterus corpus and was soft in consistency. The extent of myometrial invasion was less than 50%. On microscopy, sections from the endometrium showed an infiltrating neoplasm composed of tumor cells arranged in sheets. The individual tumor cells were round to polygonal with mild nuclear pleomorphism and had abundant clear cytoplasm, with many cells showing hobnailing features (Figure 5). Focally, the tumor was seen involving the connective tissue of the cervix. The sampled lymph nodes, omentum, bilateral fallopian tubes, and ovaries showed no tumor deposit. With the histology described above, a diagnosis of clear cell carcinoma was favored. An IHC study was performed to confirm the diagnosis. By IHC, the tumor cells were positive for Napsin A, Vimentin, and CK7. The tumor cells were negative for estrogen receptor (ER), progestrone receptor (PR) and carcinoma embryonic antigen (CEA). P53 showed strong nuclear expression of mutant type. Hence, the above findings confirmed clear cell carcinoma of the endometrium. According to the AJCC 8th edition, a stage of pT2pN0 was given with FIGO stage II. IHC was also done for DNA mismatch repair studies, where they showed intact nuclear expression and were MMR-proficient.

Case 5

A 52-year-old female had complaints of vague abdominal pain, foul-smelling discharge per vaginum, bleeding per vaginum, and bowel and bladder habits disturbances for the past few weeks (Table 1). She had three children, with the last child born 19 years ago. She has been postmenopausal for the previous three years. She has no other systemic illness. An ultrasound, both transabdominal and transvaginal, was performed revealing a lesion measuring 7 x 6 x 5 cm in the endometrial cavity. Given the above findings, staging laparotomy was done. 

**Figure 6:**
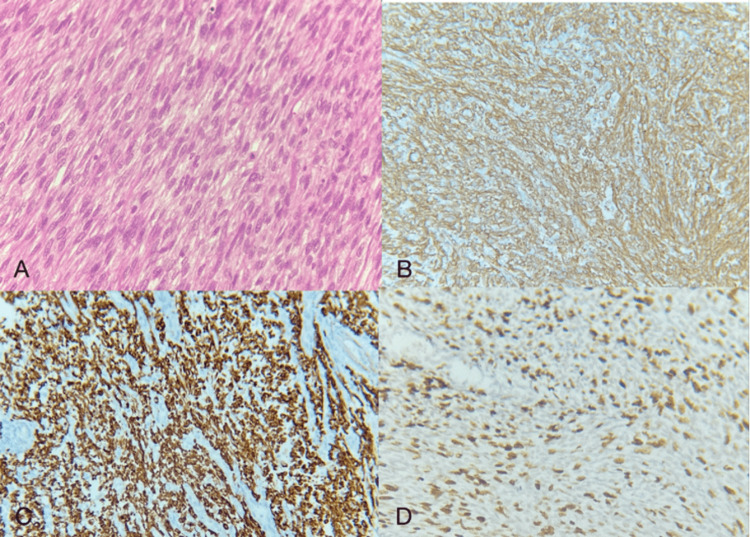
Endometrial stromal sarcoma with smooth muscle differentiation H&E: Hematoxylin and eosin; IHC: immunohistochemistry; SMA: smooth muscle actin (A) Tumor cells showing spindled morphology (40x magnification) (H&E). (B) SMA showing membranous positivity (IHC). (C) Desmin shows diffuse positivity (IHC). (D) Ki67 labelling index showed 60% expression in the tumor cells (IHC)

On gross examination, a lesion was seen arising from the fundus of the uterus which appeared grey-white in color with focal black areas. Solid and cystic areas were also seen, with 20% appearing hemorrhagic. A breach measuring 2 cm in greatest dimension was also noted in the serosal aspect. On microscopy, sections from the lesion showed tumor cells arranged in nests with individual cells having moderate eosinophilic cytoplasm, spindled-out nuclei, coarse chromatin, and scattered mitotic figures. The lesion was seen invading the myometrium, and the capsular breach confirmed the protrusion into the serosa. The ovaries and fallopian tubes showed no evidence of tumor. Since the tumor is a high-grade lesion, a stromal sarcoma was favored due to its spindled-out morphology. Immunohistochemistry showed that the tumor cells were positive for Cyclin D1, smooth muscle actin (SMA), and Desmin (Figure 6). The Ki67 labelling index was 60%. In light of the observed dual positivity of Desmin and SMA, the conclusive diagnosis points to endometrial stromal sarcoma with smooth muscle differentiation.

Case 6

**Figure 7:**
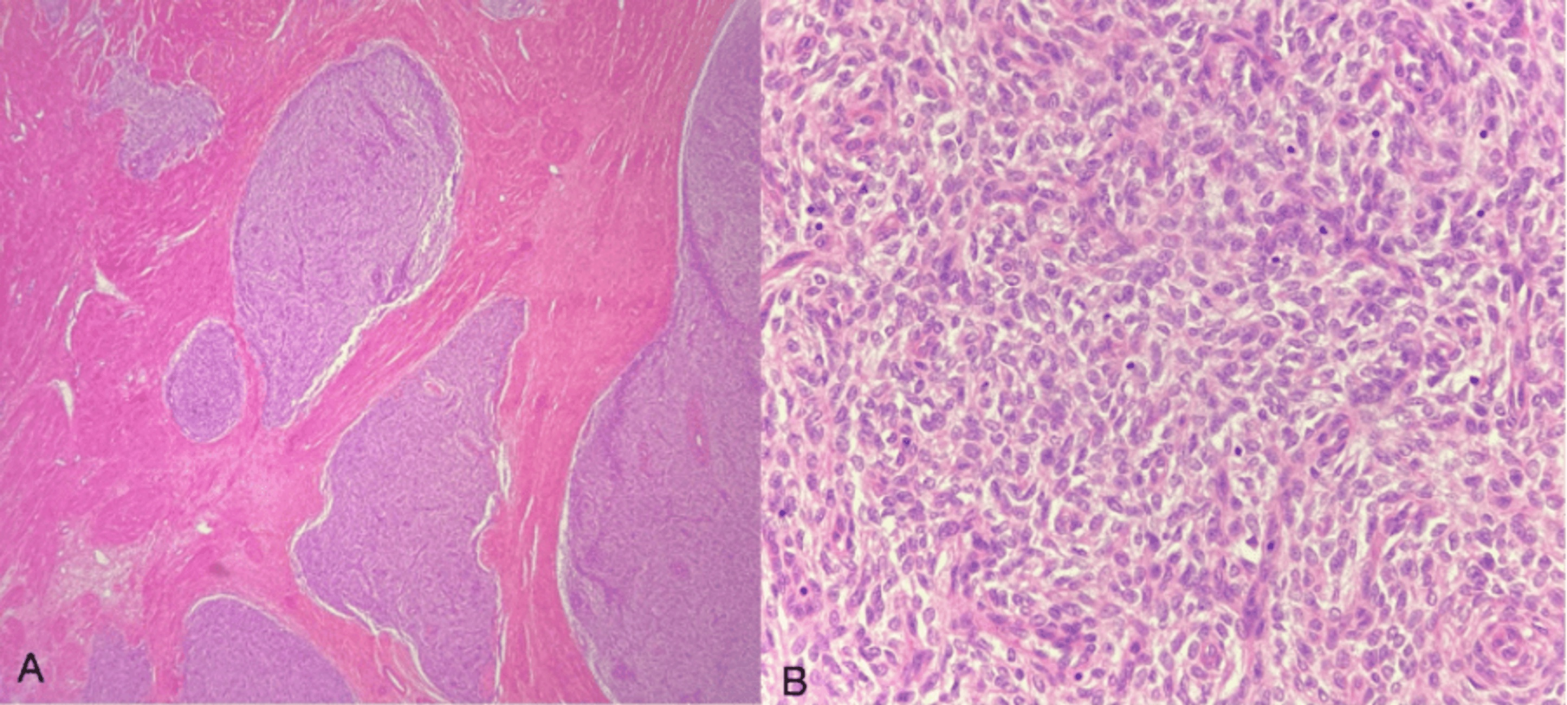
Endometrial stromal sarcoma H&E: Hematoxylin and eosin (A) Lower uterine segment showing tumor cells arranged in nests and sheets (H&E). (B) Higher magnification of the tumor cells showing indistinct cell borders and vesicular chromatin (H&E)

A 47-year-old female came to our hospital post-radical hysterectomy surgery and was diagnosed with endometrial carcinoma from another tertiary care center for review and further categorization to start the first cycle of chemotherapy (Table 1). Reviewing the histopathology specimen, a malignant infiltrating neoplasm was seen arranged in sheets, with individual cells having indistinct cell borders and vesicular nuclei (Figure 7). Lymphovascular invasion was noted, and the tumor was seen involving the lower uterine segment, right ovary, right fallopian tube, right parametrium, and left parametrium. Due to the morphology and aggressive spread of the lesion, a low-grade endometrial stromal sarcoma was given as the diagnosis. By IHC, the tumor cells were positive for CD10, and the tumor cells were negative for Cyclin D1. The Ki67 labelling index was 50% in hotspot areas. Following this diagnosis, the patient underwent chemotherapy and is currently undergoing regular follow-up.

Case 7

**Figure 8:**
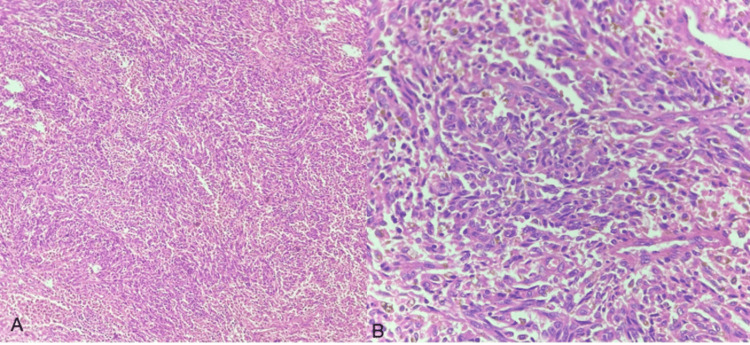
Undifferentiated sarcoma H&E: Hematoxylin and eosin (A-B) Undifferentiated sarcoma showing sheets of tumor cells with epitheloid morphology (H&E)

A 67-year-old female came with complaints of postmenopausal bleeding and radiological imaging showing multifocal lesions in the uterus and in the retroperitoneum (Table 1). The retroperitoneal mass, measuring approximately 32 x 24 x 18 cm, and the endometrial lesion, measuring 11 x 8 x 6 cm, was seen arising from the right half of the endometrial cavity. On microscopic examination, the tumor cells were seen arranged in sheets and diffusely infiltrating the endometrium, with the individual cells having an epitheloid morphology (Figure 8) and a few areas of myxoid degeneration. The tumor was graded according to the grading system by Federation National des Cenres de Lutte Contre le Cancer (FNCLCC) with a tumor differentiation of score 3, mitotic count score of 3, and tumor necrosis score of 1. No deposits were found in any of the 34 lymph nodes examined. Tumor deposits were noted in the small bowel mucosa, appendiceal epiploicae,, and bladder peritoneum. By IHC, the tumor cells were positive for Vimentin, TLE1, and CD10. They were negative for SMA, Desmin, EMA, SOX10, S100, Cyclin D1, and CD117. The Ki67 labelling index was 90%. This IHC pattern favored the diagnosis of undifferentiated sarcoma.

## Discussion

Case 1: Mesonephric-like adenocarcinoma

Mesonephric-like adenocarcinoma is usually very challenging to diagnose since it can have varied growth patterns within the same tumor and look similar to low-grade endometrioid and serous carcinoma. It has a wide range of age at presentation. The histology of this tumor is composed of small tubules/glandular growth patterns with luminal eosinophilic colloid-like secretions usually present [3]. The individual tumor cells have scant cytoplasm and show mild cytological atypia. The nuclei can be oval and sometimes shows nuclear grooves and overlapping, resembling papillary carcinoma of the thyroid [4]. There should be no associated mesonephric remnants present. These tumors are usually positive for PAX8, GATA3, and TTF-1. They are negative for ER and PR; however, some aberrant expressions can exist. Molecular pathogenesis involves the activation of the KRAS pathway and mutations in ARID1A. It displays aggressive behavior and tends to metastasize to the lungs [5]. Six key factors associated with an increased risk of metastasis include tumor size of more than 4 cm, ill-defined tumor borders, FIGO high grade, tumor cell necrosis, and high mitotic activity [5].

Although it had a better overall survival rate than carcinoma and serous carcinoma, mesonephric adenocarcinoma of the endometrium has a considerable risk of recurrent disease and a tendency to metastasize to the lungs. Hence, the patients must be kept on follow-up for it.

Cases 2 and 3: Carcinosarcoma

Carcinosarcoma is an aggressive tumor of high-grade endometrial carcinoma and shows secondary sarcomatous trans-differentiation. It is usually diagnosed at advanced stages. More than a single entity, it includes histological subtypes of carcinoma and sarcoma elements [6]. The majority of them show p53 mutation. The optimal management of patients with nonmetastatic disease consists of a stepwise approach with surgery followed by chemotherapy and radiotherapy. Carcinosarcomas are seen in postmenopausal women, and the presentation is abnormal uterine bleeding, which was also seen in these cases. Although the tumor is very aggressive, recent reports show that the overall median survival age has improved due to early diagnosis and management, making it 39 months [7].

Case 4: Clear cell carcinoma 

Clear cell carcinomas are estrogen-dependent type II endometrial carcinomas and account for less than 5% of the tumors. Microscopically, they are described as clear hobnail cells due to their rich amount of glycogen [8]. These cells can be confirmed by IHC, showing positivity for Napsin A. They tend to spread intraperitoneally and also have an increased risk of lymphovascular invasion [9]. P53 gene mutations, p16 inactivation, Her2neu overexpression, and loss of heterozygosity on several chromosomes are a few of the molecular profiles described to be associated with clear cell carcinoma. Patients presenting with clear cell carcinoma are usually of older ages, which was a similar finding in this study, and had an overall five-year survival rate of 42.3% to 62.5% in advanced stages [9]. Due to this, more aggressive adjuvant therapy is needed .

Cases 5 and 6: Endometrial stromal sarcoma

Endometrial stromal sarcoma is seen in the age group of 42-58 years, making it a challenging condition to diagnose [10]. It has a high similarity with the normal endometrium. Usually seen majorly in postmenopausal women, total abdominal hysterectomy with bilateral salpingo-ophorectomy is the treatment of choice. Ultrasound imaging for this condition is often mistaken and reported as leiomyoma, adenomyosis, or intrauterine polyps [11]. Hence, the definitive diagnostic modality is a histopathological examination with immunohistochemistry. CD10 can help distinguish these tumors from leiomyoma. The prognosis is intermediate for low-grade endometrial stromal sarcoma. Since only a limited number of case reports with follow-up information are available, there is still a need to identify the favorable and unfavorable prognostic factors. Hence, the clinicians must keep the patients on regular follow-ups.

Case 7: Undifferentiated sarcoma 

Undifferentiated sarcomas only comprise 1%-2% of all tumors from the uterine corpus. This undifferentiated tumor presents with a high mitotic count and shows an aggressive clinical course leading to death within three years of tumor dissemination [12]. Histologically, they also show myometrial invasion, severe nuclear pleomorphism, and tumor necrosis without a specific line of differentiation. Local recurrence and distant metastasis add to the increased mortality rate. Treatment is primarily surgery followed by chemotherapy and radiation [13]. Estrogen and progesterone positivity by IHC will support adjuvant hormone therapy for better response. Patients presenting at older age, having extrauterine spread, and showing evidence of tumor necrosis and increased mitosis are associated with shorter survival rates. Some molecular prognostic groups have been proposed but are not yet used in practice to stratify the patients. The median overall survival for these tumors is approximately 12-24 months.

## Conclusions

This case series showcases infrequently observed instances of endometrial carcinoma, underscoring its critical diagnostic significance and substantial impact on a patient's prognosis. Given the complex histological characteristics associated with these conditions, early detection and effective management are imperative in these scenarios. In light of evolving medical landscapes, heightened risk factors, and increased exposure to carcinogens, the rarity of these tumors may persist. A comprehensive understanding of such cases necessitates thorough research and the advancement of treatment modalities to ensure optimal patient care.
